# Type I interferon in psoriasis: mechanisms and clinical implications

**DOI:** 10.3389/fimmu.2026.1924303

**Published:** 2026-09-01

**Authors:** Chang Liu, Yidan Zhang, Xiaoqian Cui, Hailong Zhang, Jia Chen, Xiongwei Lian, Suqing Yang

**Affiliations:** Department of Dermatology, First Affiliated Hospital of Heilongjiang University of Chinese Medicine, Harbin, China

**Keywords:** IL-23/IL-17 axis, LL37, plasmacytoid dendritic cells, psoriasis, type I interferon

## Abstract

Psoriasis is a chronic, recurrent, immune-mediated inflammatory skin disease in which the TNF/IL-23/IL-17 axis plays a central role in the maintenance of mature plaques. However, this classical framework does not fully explain the early immune events triggered by skin injury, barrier disruption, infection, and the release of self-nucleic acids. It also does not fully explain selected clinical phenotypes. Type I interferons (IFN-I) are inducible cytokines involved in antiviral defense and danger sensing that may shift from protective immune mediators to pathogenic inflammatory amplifiers in psoriasis-prone skin. Self-nucleic acids released after tissue injury can form immunostimulatory complexes with antimicrobial peptides such as LL37. These complexes activate innate immune responses and induce IFN-I production. This process can promote dendritic-cell maturation and amplify the IL-23/IL-17 inflammatory pathway. This review presents IFN-I as a stage-dependent and phenotype-specific immune module that links early danger sensing to subsequent inflammatory amplification in psoriasis. We focus on the protective role of IFN-I in skin danger responses, LL37-mediated abnormal nucleic acid sensing, multicellular IFN-I skin signatures, the stage-specific integration of IFN-I with the IL-23/IL-17 axis, and clinical contexts with increased IFN-I activity. Moreover, we briefly discuss the therapeutic significance of tyrosine kinase 2 as a convergence node for IL-23, IL-12, and IFN-I signaling. Defining the role of IFN-I across different lesion stages and clinical phenotypes may improve the immunopathological model of psoriasis. It may also provide a basis for disease stratification, response prediction, and individualized therapy.

## 1 Introduction

Psoriasis is a chronic, recurrent, immune-mediated inflammatory skin disease. It is characterized by epidermal hyperplasia, abnormal keratinocyte differentiation, vascular remodeling, and infiltration of innate and adaptive immune cells (1). In recent years, the tumor necrosis factor (TNF), interleukin (IL)-23, and IL-17 pathways have become the core inflammatory axis used to explain the pathogenesis of plaque psoriasis (2). Genetic, transcriptomic, immunological, and therapeutic evidence all support this model. This is especially clear from the strong clinical efficacy of biologics targeting TNF, IL-23, IL-17A, IL-17F, and the IL-17 receptor (3). However, the IL-23/IL-17 axis mainly explains the maintenance of chronic inflammation in established lesions. It is less able to explain the upstream mechanisms by which skin injury, infection, mechanical stress, and barrier disruption are converted into early psoriasiform inflammation (4).

Type I interferons (IFN-I) provide an important perspective for understanding early immune activation in psoriasis (5). As key cytokines in antiviral defense and tissue damage sensing, IFN-I participates in pathogen clearance, epithelial immune surveillance, and the connection between innate and adaptive immunity under physiological conditions (6). However, in psoriasis-prone skin, self-DNA and self-RNA released by injury or cell death can form immunostimulatory complexes with antimicrobial peptides such as LL37 (7). These complexes can increase the immunogenicity of self-nucleic acids. They can activate plasmacytoid dendritic cells (pDCs) and keratinocytes, and induce the expression of IFN-α, IFN-β, and interferon-stimulated genes (8).

Therefore, this review examines IFN-I as a context-dependent immune program that may shift from a protective cutaneous danger response to a pathogenic inflammatory amplifier across specific stages and phenotypes of psoriasis (9). We focus on LL37-mediated self-nucleic acid sensing, pDC-derived IFN-α, keratinocyte-derived IFN-β, neutrophil extracellular trap (NET)-associated nucleic acid inflammation, and the temporal integration of IFN-I with IL-23/IL-17 signaling (10). We further discuss IFN-I-associated clinical phenotypes, tyrosine kinase 2 (TYK2)-mediated pathway crosstalk, and their implications for disease stratification and treatment selection.

## 2 IFN-I as an inducible skin danger-response program

The skin is both a physical barrier and an immune-active tissue. Keratinocytes form the epidermal barrier. They also sense environmental stress, produce cytokines and chemokines, release antimicrobial peptides, and interact with resident and recruited immune cells (11). In healthy skin, IFN-I is not expressed at a continuously high level. Instead, it is rapidly induced after viral infection, tissue injury, or barrier disruption (6).

IFN-I acts through the interferon-α/β receptor (IFNAR). It activates the Janus kinase–signal transducer and activator of transcription (JAK–STAT) pathway and induces interferon-stimulated genes (12). These genes establish an antiviral state, enhance antigen presentation, regulate chemokine production, and shape immune-cell activation. When this response is transient and matches the strength of the danger signal, it is protective. It helps the skin recognize infection or injury, restore local immune surveillance, and support tissue repair (6).

However, the same inducible defense program can become pathogenic when IFN-I is activated by self-derived nucleic acids or when its termination is impaired. This is especially important in the skin, where mechanical injury, microbial exposure, ultraviolet exposure, and barrier damage are common (5). In genetically or immunologically susceptible individuals, a normally protective IFN-I response may lower the threshold for skin inflammation. This does not mean that IFN-I alone is sufficient to produce the full psoriatic phenotype. Rather, it suggests that IFN-I may activate dendritic cells, promote chemokine gradients, and prepare the tissue environment for later adaptive immune amplification (6).

Thus, the biological meaning of IFN-I in psoriasis is highly context-dependent. Short-lived IFN-I signaling supports host defense and tissue immune surveillance (12). In contrast, persistent, repeated, or misdirected IFN-I signaling may participate in pathogenic inflammation. This may occur when abnormal antimicrobial peptide expression, self-nucleic acid release, pDC activation, and keratinocyte stress responses occur together (6, 9).

## 3 Abnormal nucleic acid sensing and multicellular IFN-I skin signatures

One key mechanism linking tissue injury to IFN-I activation in psoriasis is abnormal recognition of self-nucleic acids. Under steady-state conditions, extracellular self-DNA and self-RNA have limited immunogenicity. This is because nucleic acid-sensing receptors are located in defined intracellular compartments, and extracellular nucleases promote nucleic acid degradation (7). In psoriatic skin, however, this tolerance barrier can be broken by antimicrobial peptides, especially LL37 (8).

LL37 can bind self-DNA and self-RNA released from damaged or dying cells. This forms immunostimulatory complexes. These complexes protect nucleic acids from degradation and help deliver them to endosomal Toll-like receptors (TLRs) (7). In pDCs, LL37–self-DNA and LL37–self-RNA complexes activate TLR9 and TLR7, respectively, whereas TLR8-mediated responses occur mainly in myeloid dendritic cells (7, 8). Through this process, self-nucleic acids are no longer passive products of tissue injury. They become danger signals that can initiate innate immune activation.

Beyond its role as a carrier of self-nucleic acids, LL37 can also function as a T-cell autoantigen in psoriasis. LL37-specific CD4+ and/or CD8+ T cells have been detected in approximately two-thirds of patients with moderate-to-severe plaque psoriasis. These cells produce IFN-γ, while LL37-reactive CD4+ T cells also produce Th17 cytokines, indicating a mixed Th1/Th17 phenotype. LL37-specific T cells can infiltrate psoriatic lesions, and their circulating frequency is associated with disease activity. Thus, LL37 may link nucleic acid-driven innate immune activation with antigen-specific adaptive immunity (13).

pDCs are among the strongest producers of IFN-I. They are scarce in steady-state skin and are recruited predominantly to the dermis, including the dermal papillae, in early psoriatic lesions (5, 9). In contrast, Langerhans cells constitute the principal antigen-presenting cell population resident in the epidermis (14). Activation of pDCs in pre-psoriatic skin or early lesions supports the view that innate immune responses may occur before a full adaptive T-cell response is established. pDC-derived IFN-α can promote the maturation of conventional dendritic cells, enhance antigen presentation, and create an inflammatory microenvironment that supports later IL-23 production and IL-17-producing T-cell responses (10).

Keratinocytes add a second layer to this model. They are not only target cells of IL-17-driven inflammation. They are also active immune-regulatory cells and potential contributors to IFN-I skin signatures (11). In response to injury, microbial products, endogenous RNA, and LL37-related nucleic acid complexes, keratinocytes can produce IFN-β and induce the expression of interferon-stimulated genes (15). This extends the traditional pDC-centered model of IFN-I biology in psoriasis. It suggests that epithelial cells themselves may also participate in the local IFN-I program. Keratinocytes can also respond to IFN-I by producing chemokines, antimicrobial peptides, and inflammatory mediators. This can amplify immune-cell recruitment and epidermal remodeling (11).

Neutrophils and NETs may further amplify nucleic acid-driven inflammation in selected contexts. NETs contain DNA, RNA, histones, granule proteins, and antimicrobial peptides, including LL37 (16). They can therefore provide both nucleic acid ligands and carrier molecules that maintain immune activation. This mechanism may be more relevant to neutrophil-rich, highly active, acute, or pustular phenotypes (17). It should not be generalized to all chronic plaque psoriasis lesions. The relationship among NETs, LL37, IL-36, and IFN-I should be understood as a phenotype-associated inflammatory amplification loop, rather than a universal mechanism in all psoriatic lesions.

Overall, IFN-I skin signatures in psoriasis should be understood as the result of multicellular epithelial–immune interactions (11). pDCs, keratinocytes, neutrophils, dendritic cells, and T cells do not act in isolation. Their interactions determine whether a short-lived danger response resolves or develops into persistent psoriatic inflammation (2).

## 4 Stage-specific integration of IFN-I with the IL-23/IL-17 axis

IFN-I and the IL-23/IL-17 axis represent temporally linked components of a dynamic inflammatory cascade (10). In early or injury-related contexts, IFN-I may function as an upstream immune alarm signal by activating dendritic cells, enhancing antigen presentation, shaping chemokine networks, and promoting IL-23 production. These effects can support the recruitment, activation, and polarization of pathogenic IL-17-producing cells. Once the IL-23/IL-17 axis is established, IL-17 and IL-22 act on keratinocytes and induce antimicrobial peptides, chemokines, inflammatory cytokines, and epidermal proliferative responses (18, 19). This establishes a self-sustaining inflammatory loop characteristic of mature psoriatic plaques.

This temporal model helps reconcile apparently conflicting observations. IFN-I can induce or aggravate psoriasiform inflammation during IFN-α therapy, skin injury, TLR activation, or TNF blockade (10), whereas direct inhibition of IL-17 or IL-23 is highly effective in established plaque psoriasis (3). These observations place IFN-I predominantly in lesion initiation and selected IFN-I-high states, while emphasizing the dependence of mature plaques on downstream effector pathways.

**Figure 1:**
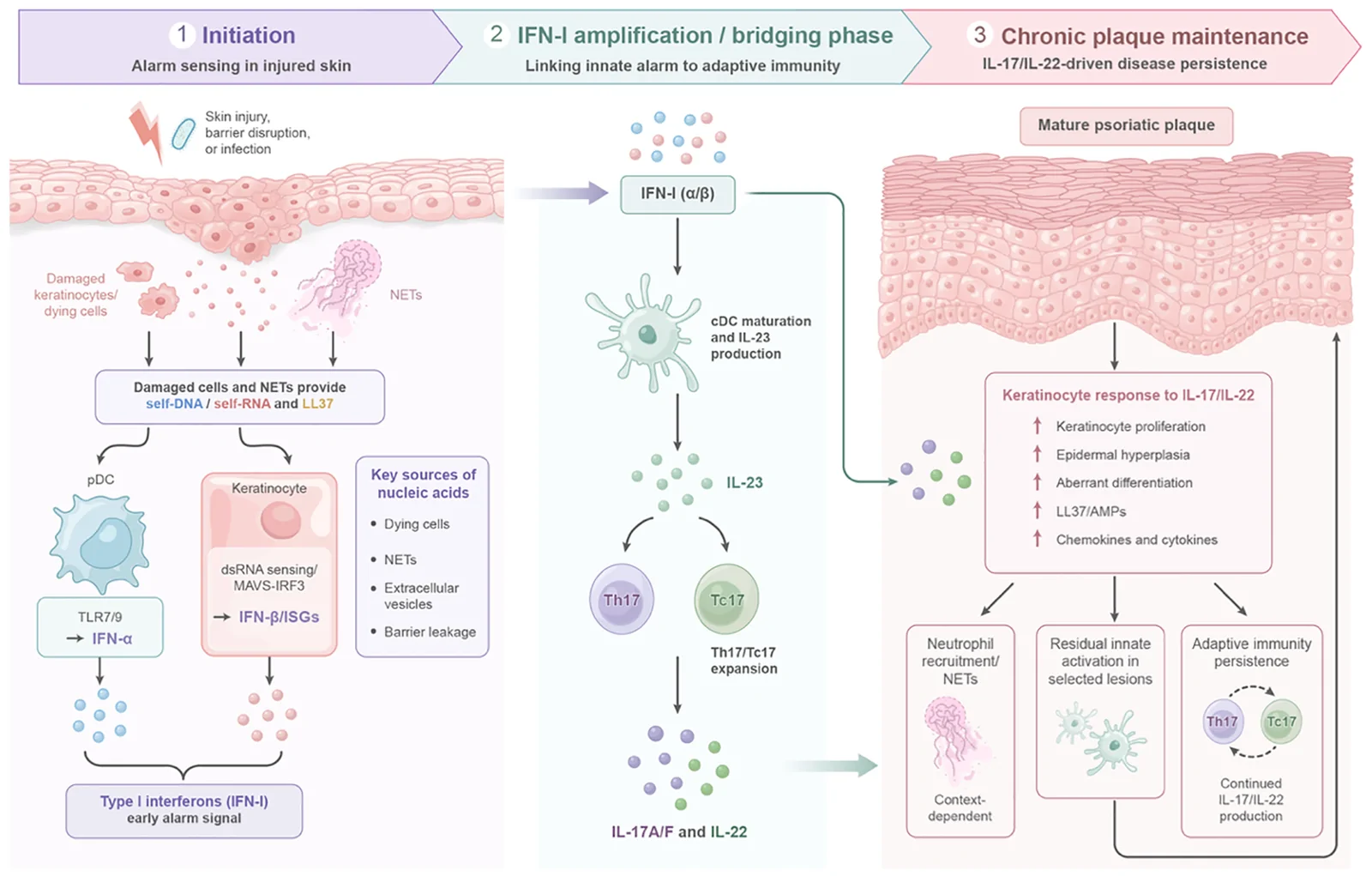
Stage-specific role of type I interferons in psoriasis initiation and inflammatory amplification. Skin injury, infection, mechanical stress, or barrier disruption can lead to the release of self-DNA and self-RNA from damaged cells, extracellular vesicles, and neutrophil extracellular traps. In psoriasis-prone skin, self-nucleic acids may form immunostimulatory complexes with antimicrobial peptides such as LL37, thereby facilitating immune recognition of extracellular nucleic acids. These complexes activate nucleic acid-sensing pathways in plasmacytoid dendritic cells, particularly TLR7/9-dependent signaling, leading to IFN-α production. In parallel, injured or stressed keratinocytes may activate cytosolic dsRNA-sensing pathways, including RIG-I/MDA5–MAVS–IRF3 signaling, and produce IFN-β and interferon-stimulated genes. NETs may further amplify nucleic acid-driven inflammation in neutrophil-rich or highly active lesions. IFN-I can promote conventional dendritic-cell maturation, antigen presentation, IL-23 production, and subsequent Th17/Tc17 responses with IL-17/IL-22 release. Thus, IFN-I mainly contributes to early immune activation and selected inflammatory contexts, whereas established plaques are driven predominantly by IL-23/IL-17-mediated keratinocyte activation and tissue-resident immune networks. AMPs, antimicrobial peptides; cDCs, conventional dendritic cells; dsRNA, double-stranded RNA; IFN-I, type I interferon; IFN-α, interferon-α; IFN-β, interferon-β; IL, interleukin; IRF3, interferon regulatory factor 3; ISGs, interferon-stimulated genes; MAVS, mitochondrial antiviral signaling protein; MDA5, melanoma differentiation-associated protein 5; NETs, neutrophil extracellular traps; pDCs, plasmacytoid dendritic cells; RIG-I, retinoic acid-inducible gene I; Tc17, IL-17-producing cytotoxic T cells; Th17, T helper 17 cells; TLR, Toll-like receptor.

The distinction between initiation and maintenance also has implications for disease recurrence. Clinically resolved psoriatic skin can retain local immune memory, including tissue-resident T cells and altered stromal or epithelial states (20). Lesion recurrence may therefore rely less on the initial IFN-I-associated program and more on tissue-resident immune networks and IL-23/IL-17-dependent recall responses (21). Lesion duration, anatomical site, treatment exposure, disease phenotype, and biopsy timing should consequently be considered when interpreting IFN-I signatures. Thus, IFN-I provides a mechanistic link between tissue injury, abnormal nucleic acid sensing, early innate immune activation, and subsequent IL-23/IL-17-driven inflammatory amplification (**Figure 1**).

## 5 Clinical phenotypes with increased IFN-I activity and therapeutic implications

**Figure 2:**
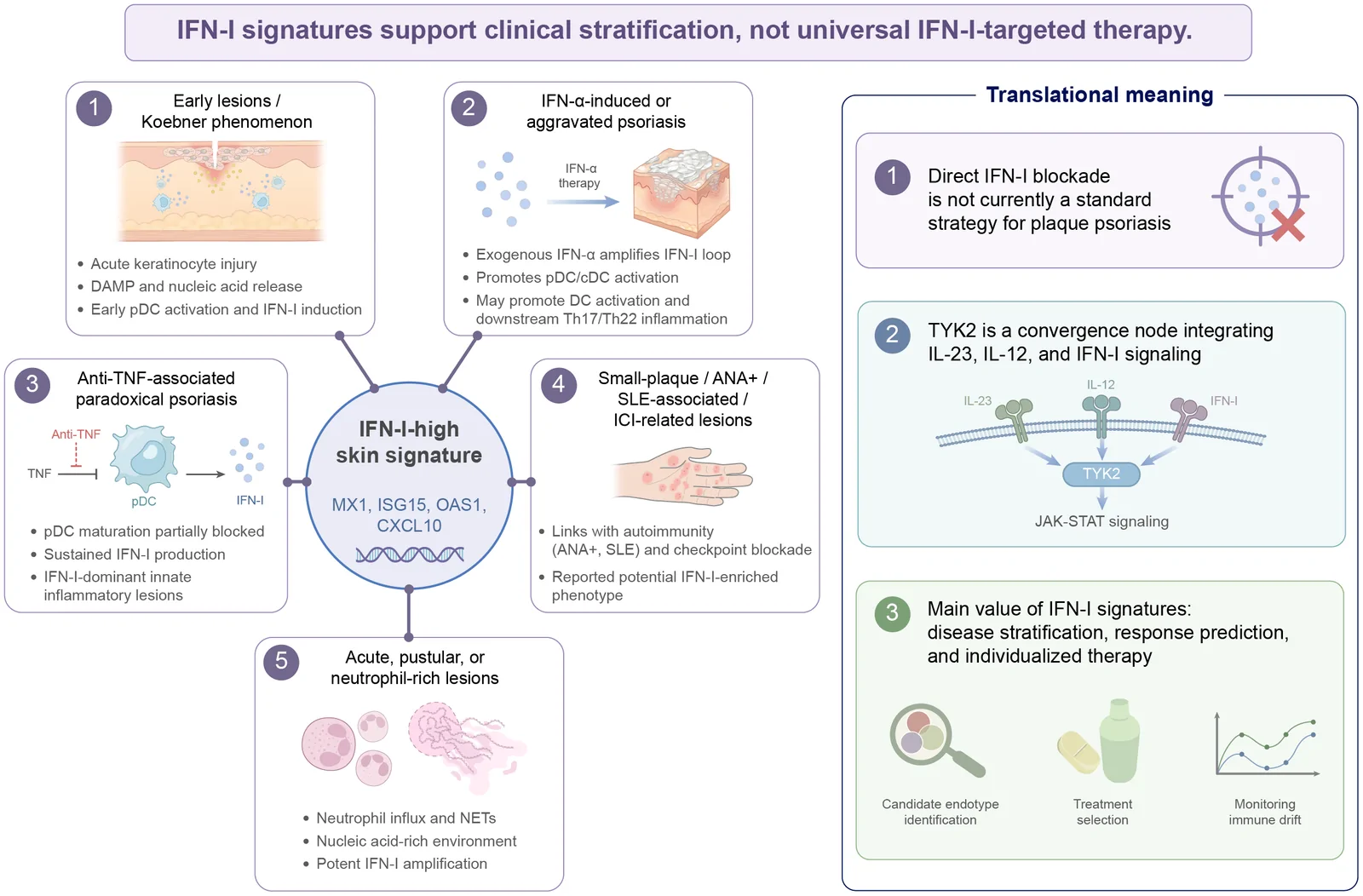
IFN-I skin signatures in psoriasis: clinical contexts and translational implications. IFN-I -related skin signatures may be enriched in selected clinical contexts rather than representing a universal mechanism across all plaque psoriasis lesions. These contexts include early or Koebner-associated lesions, IFN-α-induced or aggravated psoriasis, anti-TNF-associated paradoxical psoriasis, small-plaque, ANA-positive, SLE-associated or ICI-related lesions, and acute, pustular, or neutrophil-rich lesions. These settings may involve increased self-nucleic acid release or sensing, pDC/cDC activation, keratinocyte stress responses, NET-associated inflammation, and sustained IFN-I-related transcriptional programs. However, direct IFN-I blockade is not currently a standard therapeutic strategy for plaque psoriasis. The main translational value of IFN-I signatures may lie in candidate endotype identification, clinical stratification, potential response prediction, monitoring of immune shifts, and pathway-informed individualized therapy. TYK2 represents a convergence node integrating IL-23, IL-12, and IFN-I signaling, highlighting pathway crosstalk rather than selective IFN-I blockade. ANA, antinuclear antibody; cDCs, conventional dendritic cells; CXCL10, C-X-C motif chemokine ligand 10; ICI, immune checkpoint inhibitor; IFN-I, type I interferon; IFN-α, interferon-α; IL, interleukin; ISG15, interferon-stimulated gene 15; JAK–STAT, Janus kinase–signal transducer and activator of transcription; MX1, MX dynamin-like GTPase 1; NETs, neutrophil extracellular traps; OAS1, 2′-5′-oligoadenylate synthetase 1; pDCs, plasmacytoid dendritic cells; SLE, systemic lupus erythematosus; Th17, T helper 17 cells; Th22, T helper 22 cells; TNF, tumor necrosis factor; TYK2, tyrosine kinase 2.

Several clinical observations support the relevance of IFN-I in psoriasiform inflammation. IFN-α therapy can induce new-onset psoriasis or aggravate existing psoriasis (22). This suggests that excessive IFN-I signaling may lower the threshold for skin inflammation in susceptible individuals. Rather than acting as an independent and universal disease driver, IFN-I may have a permissive or amplifying role in specific genetic, immune, or treatment-related settings (6) (**Figure 2**).

TNF inhibitor-associated paradoxical psoriasis is one of the clearest clinical models of IFN-I dysregulation. Although TNF inhibitors are effective in classical psoriasis and other immune-mediated diseases, some patients develop psoriasiform lesions during TNF blockade (23). TNF usually promotes pDC maturation and limits sustained IFN-I production. Its blockade may impair pDC maturation, sustain IFN-I release, and prolong innate immune activation (24). This explains why paradoxical psoriasis may resemble classical psoriasis clinically while showing a distinct immunological profile. It also supports the concept that similar cutaneous phenotypes may arise from different dominant inflammatory modules.

Small-plaque psoriasis may represent another clinical context with increased IFN-I activity. Increased expression of IFN-I-related markers, such as myxovirus resistance protein A, LL37, and IL-36γ, has been reported in small-plaque lesions associated with anti-TNF therapy, systemic lupus erythematosus or antinuclear antibody positivity, and immune checkpoint inhibitor therapy (25). This suggests that lesion morphology may partly reflect the underlying immune pathway in selected settings. However, the available evidence is based on limited sample sizes. Small-plaque psoriasis should therefore be regarded as a hypothesis-generating phenotype rather than a confirmed IFN-I-driven subtype.

Acute, pustular, neutrophil-rich, or highly active lesions may also have a stronger IFN-I-related nucleic acid inflammatory component. This may be especially true when NETs, LL37, IL-36, and innate immune activation are prominent (16). In contrast, chronic stable plaques may depend more on IL-23/IL-17-driven keratinocyte activation and tissue-resident immune responses (20). This distinction is important for biomarker development. An IFN-I signature detected in a single lesion or at a single time point may not represent the dominant biology of the whole disease course.

Taken together, these clinical contexts do not support direct IFN-I blockade as a standard treatment strategy for plaque psoriasis. The marked efficacy of IL-17 and IL-23-targeted biologics confirms that downstream effector pathways remain central to the maintenance of established plaques (3). The therapeutic relevance of IFN-I may therefore lie primarily in phenotype stratification, treatment-related immune shifts, and the identification of context-specific inflammatory programs rather than in direct pathway inhibition (6).

TYK2 mediates signaling downstream of IL-12, IL-23, and IFN-I receptors, and selective TYK2 inhibition consequently modulates multiple inflammatory pathways (26). In established plaque psoriasis, the clinical efficacy of deucravacitinib is generally interpreted primarily in the context of IL-23/Th17 pathway suppression. Because multiple TYK2-dependent cytokine pathways are inhibited concurrently, current clinical evidence does not permit the therapeutic effects of TYK2 inhibition to be attributed specifically to suppression of IFN-I signaling.

Among TYK2 inhibitors, the strongest clinical evidence is available for deucravacitinib, an oral selective allosteric TYK2 inhibitor. An earlier phase 2 trial demonstrated its efficacy and tolerability (27). In the phase 3 POETYK PSO-1 and PSO-2 trials, deucravacitinib was more effective than placebo and apremilast at week 16, with clinical benefits maintained through week 52 (28, 29). Four-year extension data further showed durable efficacy, an overall stable safety profile, and no new safety signals (30). In the 16-week phase 4 IMMpactful trial, risankizumab achieved higher PASI 90 and PASI 100 response rates than deucravacitinib, while neither treatment showed new or unexpected safety signals (31). These findings support deucravacitinib as a long-term oral treatment option and indicate greater short-term skin clearance with risankizumab. However, medium- to long-term head-to-head efficacy and safety data remain limited.

Precision dermatology provides a useful framework for integrating these findings. Inflammatory skin diseases can be classified according to dominant immune modules, including Th17, Th2, Th1, IFN-I, and neutrophil/IL-1/IL-36 programs (32). Within this framework, psoriasis should not be viewed as a single molecular entity across all clinical contexts. IFN-I signatures may help identify patients or lesions in which upstream innate immune activation, paradoxical pathway switching, or treatment-induced immune drift contributes to disease expression (23). Future studies should determine whether IFN-I-related signatures have independent value for phenotype stratification or treatment selection. Any association between IFN-I signatures and response to TYK2 inhibition should be evaluated alongside IL-23/Th17-related biomarkers, because treatment response cannot be attributed to IFN-I suppression alone (**Supplementary Table 1**).

## 6 Unresolved questions and future directions

Several key questions remain unresolved. First, the temporal dynamics of IFN-I during psoriasis development need to be clarified. Longitudinal sampling of non-lesional skin, pre-lesional skin, early lesions, established plaques, resolved plaques, and recurrent lesions may help distinguish IFN-I programs related to lesion initiation from immune loops related to inflammation maintenance (20).

Second, the spatial relationship between IFN-I-producing cells and IFN-I-responsive cells requires further study. Single-cell transcriptomics and spatial transcriptomics may help define whether IFN-I signals mainly arise from pDCs, keratinocytes, neutrophil-rich areas, or specific epithelial–immune niches (33). These methods may also clarify how IFN-I-responsive keratinocyte states interact with dendritic cells and IL-17-producing lymphocytes (34).

Third, IFN-I signature detection needs to be standardized before clinical translation. Different studies have used different indicators, including IFN-α, IFN-β, myxovirus resistance protein A, ISG15, OAS family genes, CXCL10, and broader interferon-stimulated gene modules (6). Future biomarker studies should establish reproducible IFN-I signature panels. They should also assess their relationships with lesion stage, morphology, treatment history, biopsy site, and clinical outcomes (25).

Fourth, IFN-I-high phenotypes need prospective validation. Paradoxical psoriasis, IFN-α-induced psoriasis, small-plaque psoriasis, immune checkpoint inhibitor-related psoriasiform eruptions, and acute or neutrophil-rich lesions are all important clinical contexts (22, 25). However, they still need to be studied using unified transcriptomic, histological, and treatment-response endpoints (32).

Finally, IFN-I biology should be integrated into a broader immune-module model of psoriasis. It should not be studied in isolation (32). Such a model may help define psoriasis as a dynamic disease. Its dominant inflammatory program may change over time and may be influenced by lesion stage, treatment exposure, and host background (20).

## 7 Conclusion

IFN-I signaling in psoriasis reflects the participation of a protective host defense program in pathogenic inflammation under a susceptible tissue background. Under physiological conditions, IFN-I supports antiviral defense, tissue danger sensing, and immune coordination. However, in psoriasis-prone skin, injury-induced release of self-nucleic acids, LL37-mediated nucleic acid complex formation, pDC activation, keratinocyte stress responses, and NET-associated inflammatory amplification may together shift this protective program toward inflammation initiation and expansion.

IFN-I is particularly relevant to lesion initiation and selected phenotype-specific contexts, including the Koebner phenomenon, disease flares, IFN-α-induced psoriasis, TNF inhibitor-associated paradoxical psoriasis, and certain morphological or transcriptomic phenotypes with increased IFN-I activity. In contrast, mature chronic plaques remain predominantly maintained by IL-23/IL-17-driven keratinocyte activation and tissue-resident immune networks. A stage-specific and phenotype-sensitive understanding of IFN-I may improve the immunopathological model of psoriasis and support more precise clinical stratification. Future studies should further distinguish the functional roles of IFN-I across different disease stages and clinical phenotypes. They should clarify whether IFN-I acts as an inflammatory driver, an accompanying molecular marker, or a treatment-related immune module.

## References

[1] Griffiths CEM Armstrong AW Gudjonsson JE Barker JNWN . Psoriasis. Lancet. (2021) 397:1301–15. doi: 10.1016/S0140-6736(20)32549-6 33812489

[2] Lowes MA Suárez-Fariñas M Krueger JG . Immunology of psoriasis. Annu Rev Immunol. (2014) 32:227–55. doi: 10.1146/annurev-immunol-032713-120225 24655295 PMC4229247

[3] Armstrong AW Read C . Pathophysiology, clinical presentation, and treatment of psoriasis: a review. JAMA. (2020) 323:1945–60. doi: 10.1001/jama.2020.4006 32427307

[4] Zhang LJ . Type 1 interferons potential initiating factors linking skin wounds with psoriasis pathogenesis. Front Immunol. (2019) 10:1440. doi: 10.3389/fimmu.2019.01440 31293591 PMC6603083

[5] Nestle FO Conrad C Tun-Kyi A Homey B Gombert M Boyman O . Plasmacytoid predendritic cells initiate psoriasis through interferon-alpha production. J Exp Med. (2005) 202:135–43. doi: 10.1084/jem.20050500 15998792 PMC2212894

[6] Hile GA Gudjonsson JE Kahlenberg JM . The influence of interferon on healthy and diseased skin. Cytokine. (2020) 132:154605. doi: 10.1016/j.cyto.2018.11.022 30527631 PMC6551332

[7] Lande R Gregorio J Facchinetti V Chatterjee B Wang YH Homey B . Plasmacytoid dendritic cells sense self-DNA coupled with antimicrobial peptide. Nature. (2007) 449:564–9. doi: 10.1038/nature06116 17873860

[8] Ganguly D Chamilos G Lande R Gregorio J Meller S Facchinetti V . Self-RNA–antimicrobial peptide complexes activate human dendritic cells through TLR7 and TLR8. J Exp Med. (2009) 206:1983–94. doi: 10.1084/jem.20090480 19703986 PMC2737167

[9] Grine L Dejager L Libert C Vandenbroucke RE . An inflammatory triangle in psoriasis: TNF, type I IFNs and IL-17. Cytokine Growth Factor Rev. (2015) 26:25–33. doi: 10.1016/j.cytogfr.2014.10.009 25434285

[10] Wang WM Li F Jin HZ . Role of interferon regulatory factor-mediated signaling in psoriasis. Int J Med Sci. (2021) 18:3794–9. doi: 10.7150/ijms.61973 34790055 PMC8579288

[11] Klimitz FJ Shen Y Repetto F Brown S Knoedler L Ko CJ . Keratinocytes as active regulators of cutaneous and mucosal immunity: a systematic review across inflammatory epithelial disorders. Front Immunol. (2025) 16:1694066. doi: 10.3389/fimmu.2025.1694066 41479908 PMC12753988

[12] Barrat FJ Crow MK Ivashkiv LB . Interferon target-gene expression and epigenomic signatures in health and disease. Nat Immunol. (2019) 20:1574–83. doi: 10.1038/s41590-019-0466-2 31745335 PMC7024546

[13] Lande R Botti E Jandus C Dojcinovic D Fanelli G Conrad C . The antimicrobial peptide LL37 is a T-cell autoantigen in psoriasis. Nat Commun. (2014) 5:5621. doi: 10.1038/ncomms6621 25470744

[14] Haniffa M Gunawan M Jardine L . Human skin dendritic cells in health and disease. J Dermatol Sci. (2015) 77:85–92. doi: 10.1016/j.jdermsci.2014.08.012 25301671 PMC4728191

[15] Zhang LJ Sen GL Ward NL Johnston A Chun K Chen Y . Antimicrobial peptide LL37 and MAVS signaling drive interferon-β production by epidermal keratinocytes during skin injury. Immunity. (2016) 45:119–30. doi: 10.1016/j.immuni.2016.06.021 27438769 PMC4957248

[16] Herster F Bittner Z Archer NK Dickhöfer S Eisel D Eigenbrod T . Neutrophil extracellular trap-associated RNA and LL37 enable self-amplifying inflammation in psoriasis. Nat Commun. (2020) 11:105. doi: 10.1038/s41467-019-13756-4 31913271 PMC6949246

[17] Shao S Fang H Dang E Xue K Zhang J Li B . Neutrophil extracellular traps promote inflammatory responses in psoriasis via activating epidermal TLR4/IL-36R crosstalk. Front Immunol. (2019) 10:746. doi: 10.3389/fimmu.2019.00746 31024570 PMC6460719

[18] Lowes MA Russell CB Martin DA Towne JE Krueger JG . The IL-23/T17 pathogenic axis in psoriasis is amplified by keratinocyte responses. Trends Immunol. (2013) 34:174–81. doi: 10.1016/j.it.2012.11.005 23291100 PMC3721313

[19] Furue M Furue K Tsuji G Nakahara T . Interleukin-17A and keratinocytes in psoriasis. Int J Mol Sci. (2020) 21:1275. doi: 10.3390/ijms21041275 32070069 PMC7072868

[20] Puig L Costanzo A Muñoz-Elías EJ Jazra M Wegner S Paul C . The biological basis of disease recurrence in psoriasis: a historical perspective and current models. Br J Dermatol. (2022) 186:773–81. doi: 10.1111/bjd.20963 34939663 PMC9374062

[21] Blauvelt A . Resident memory T cells in psoriasis: key to a cure? J Psoriasis Psoriatic Arthritis. (2022) 7:157–9. doi: 10.1177/24755303221127338 39296964 PMC11361500

[22] Afshar M Martinez AD Gallo RL Hata TR . Induction and exacerbation of psoriasis with interferon-alpha therapy for hepatitis C: a review and analysis of 36 cases. J Eur Acad Dermatol Venereol. (2013) 27:771–8. doi: 10.1111/j.1468-3083.2012.04582.x 22671985 PMC3443510

[23] Conrad C Di Domizio J Mylonas A Belkhodja C Demaria O Navarini AA . TNF blockade induces a dysregulated type I interferon response without autoimmunity in paradoxical psoriasis. Nat Commun. (2018) 9:25. doi: 10.1038/s41467-017-02466-4 29295985 PMC5750213

[24] Mylonas A Conrad C . Psoriasis: classical vs. paradoxical. The Yin-Yang of TNF and type I interferon. Front Immunol. (2018) 9:2746. doi: 10.3389/fimmu.2018.02746 30555460 PMC6283263

[25] Khosravi-Hafshejani T Ghoreishi M Vera Kellet C Crawford RI Martinka M Dutz JP . Small plaque psoriasis re-visited: a type of psoriasis mediated by a type-I interferon pathway. Exp Dermatol. (2022) 31:753–63. doi: 10.1111/exd.14513 34890074

[26] Krueger JG McInnes IB Blauvelt A . Tyrosine kinase 2 and Janus kinase–signal transducer and activator of transcription signaling and inhibition in plaque psoriasis. J Am Acad Dermatol. (2022) 86:148–57. doi: 10.1016/j.jaad.2021.06.869 34224773

[27] Papp K Gordon K Thaçi D Morita A Gooderham M Foley P . Phase 2 trial of selective tyrosine kinase 2 inhibition in psoriasis. N Engl J Med. (2018) 379:1313–21. doi: 10.1056/NEJMoa1806382 30205746

[28] Armstrong AW Gooderham M Warren RB Papp KA Strober B Thaci D . Deucravacitinib versus placebo and apremilast in moderate to severe plaque psoriasis: efficacy and safety results from the 52-week, randomized, double-blinded, placebo-controlled phase 3 POETYK PSO-1 trial. J Am Acad Dermatol. (2023) 88:29–39. doi: 10.1016/j.jaad.2022.07.002 35820547

[29] Strober B Thaçi D Sofen H Kircik L Gordon KB Foley P . Deucravacitinib versus placebo and apremilast in moderate to severe plaque psoriasis: efficacy and safety results from the 52-week, randomized, double-blinded, phase 3 Program fOr Evaluation of TYK2 inhibitor psoriasis second trial. J Am Acad Dermatol. (2023) 88:40–51. doi: 10.1016/j.jaad.2022.08.061 36115523

[30] Armstrong AW Lebwohl M Warren RB Sofen H Morita A Paul C . Deucravacitinib in plaque psoriasis: four-year safety and efficacy results from the phase 3 POETYK PSO-1, PSO-2 and long-term extension trials. J Eur Acad Dermatol Venereol. (2025) 39:1336–51. doi: 10.1111/jdv.20553 40045918 PMC12188513

[31] Magnolo N Soung J Frew J Costanzo A Ruiz-Santiago H Eyerich K . Risankizumab versus deucravacitinib in adults with moderate plaque psoriasis: 16-week results from the phase 4 IMMpactful trial. Dermatol Ther (Heidelb). (2026) 16:3415–29. doi: 10.1007/s13555-026-01779-x 42143644 PMC13280295

[32] Di Domizio J Girardin A Conrad C Seremet T Gilliet M . Dermatology 2.0: precision medicine for inflammatory skin diseases. J Eur Acad Dermatol Venereol. (2026) 40:440–5. doi: 10.1111/jdv.70251 41453833 PMC12933700

[33] Hughes TK Wadsworth MI Gierahn TM Do T Weiss D Andrade PR . Second-strand synthesis-based massively parallel scRNA-seq reveals cellular states and molecular features of human inflammatory skin pathologies. Immunity. (2020) 53:878–894.e7. doi: 10.1016/j.immuni.2020.09.015 33053333 PMC7562821

[34] Ma F Plazyo O Billi AC Tsoi LC Xing X Wasikowski R . Single cell and spatial sequencing define processes by which keratinocytes and fibroblasts amplify inflammatory responses in psoriasis. Nat Commun. (2023) 14:3455. doi: 10.1038/s41467-023-39020-4 37308489 PMC10261041

