# Supplementary material for: Type I interferon in psoriasis: mechanisms and clinical implications
Source: Front Immunol. 2026 Sep 1;17:1924303. doi: 10.3389/fimmu.2026.1924303 (PMC13575738; doi:10.3389/fimmu.2026.1924303)
Supplement: Supplementary file 1 [file Table1.docx]

**Supplementary material**

**STable 1. Stage- and phenotype-specific interpretation of IFN-I activity in psoriasis.**

| **Context** | **IFN-I-related evidence or feature** | **Proposed role of IFN-I** | **Relationship with IL-23/IL-17 axis** | **Translational implication** | **Key references** |
| --- | --- | --- | --- | --- | --- |
| Healthy skin or transient danger response | IFN-I is rapidly induced after viral infection, tissue injury, or barrier disruption. | Protective immune alarm and epithelial immune surveillance. | Not directly pathogenic when transient and properly resolved. | Provides the physiological background for understanding IFN-I as a danger-response program. | [6] |
| Skin injury, barrier disruption, or Koebner-associated early lesions | Self-DNA/RNA released from damaged cells can form immunostimulatory complexes with LL37 and activate nucleic acid sensing. | Early upstream immune alarm; may lower the threshold for psoriasiform inflammation in susceptible skin. | May promote dendritic-cell activation and prepare the tissue environment for later IL-23/IL-17 amplification. | Helps explain why nonspecific skin injury can trigger lesion formation in predisposed individuals. | [4, 7] |
| pDC-dominant early innate activation | pDCs can be recruited to pre-psoriatic or early inflamed skin and produce IFN-α. | Initiating or permissive factor in early innate immune activation. | Supports conventional dendritic-cell maturation and later IL-23/IL-17 responses. | Useful for understanding early lesion formation, but not necessarily chronic plaque maintenance. | [5] |
| Keratinocyte IFN-I response | Keratinocytes can produce IFN-β and interferon-stimulated genes after injury or nucleic acid-related stimulation. | Epithelial amplification of local IFN-I signatures. | May enhance chemokine production, antimicrobial peptide expression, and immune-cell recruitment. | Supports the view that keratinocytes are active immune regulators, not only IL-17 target cells. | [13] |
| NET-associated nucleic acid inflammation | NETs provide DNA/RNA, LL37, histones, and granule proteins that may amplify local inflammation. | Context-dependent inflammatory amplifier. | May strengthen innate inflammation and interact with IL-36 and IL-17-related pathways. | More relevant to neutrophil-rich, acute, highly active, or pustular phenotypes than to all stable plaques. | [14, 15] |
| Established chronic plaque psoriasis | Mature plaques show strong dependence on IL-23/IL-17-driven keratinocyte activation. | IFN-I is not considered a universal dominant driver. | IL-23/IL-17 axis remains the main maintenance pathway. | Direct IFN-I blockade is not supported as a standard strategy for plaque psoriasis. | [16, 17] |
| Resolved or recurrent lesions | Clinically resolved skin may retain tissue-resident immune memory and altered tissue states. | IFN-I may be less central in recurrence than in initial lesion formation. | IL-23/IL-17-dependent recall responses and tissue-resident immune networks may dominate. | IFN-I signatures should be interpreted according to lesion stage and biopsy timing. | [18] |
| IFN-α-induced or aggravated psoriasis | IFN-α therapy can induce new-onset psoriasis or aggravate existing disease. | Permissive or amplifying factor in susceptible individuals. | May facilitate downstream psoriasiform immune activation. | Supports the concept that excessive IFN-I can lower the inflammatory threshold. | [20] |
| TNF inhibitor-associated paradoxical psoriasis | TNF blockade can lead to dysregulated IFN-I responses and paradoxical psoriasiform lesions. | IFN-I-dominant treatment-related immune module. | Distinct from classical TNF/IL-23/IL-17-dominant plaque psoriasis. | Important model for treatment-induced pathway switching. | [21] |
| Small-plaque or autoimmunity-associated lesions | Small-plaque lesions may show increased IFN-I-related markers, including myxovirus resistance protein A, LL37, and IL-36γ. | Possible IFN-I-high phenotype. | May coexist with, rather than replace, IL-23/IL-17-related inflammation. | Hypothesis-generating phenotype; needs prospective validation. | [23] |
| TYK2-related therapeutic context | TYK2 participates in signaling downstream of IL-23, IL-12, and IFN-I receptors. | Pathway convergence node rather than a selective IFN-I target. | Connects IL-23/Th17 and IFN-I-related inflammatory programs. | May support pathway-informed treatment selection and response prediction. | [24] |
| Precision stratification and future biomarkers | IFN-I signatures may vary by lesion stage, morphology, treatment history, and immune module. | Candidate molecular marker rather than a standalone disease mechanism. | Should be interpreted together with Th17, Th1, Th2, and neutrophil/IL-36 modules. | May support candidate endotype identification, immune-drift monitoring, and individualized therapy. | [27] |

IFN-I, type I interferons; IL, interleukin; NETs, neutrophil extracellular traps; pDCs, plasmacytoid dendritic cells; Th, T helper; TNF, tumor necrosis factor; TYK2, tyrosine kinase 2.
